# Service Level Factors Associated with Cervical Screening in Aboriginal and Torres Strait Islander Primary Health Care Centres in Australia

**DOI:** 10.3390/ijerph16193630

**Published:** 2019-09-27

**Authors:** Abbey Diaz, Brenda Vo, Peter D. Baade, Veronica Matthews, Barbara Nattabi, Jodie Bailie, Lisa J. Whop, Ross Bailie, Gail Garvey

**Affiliations:** 1 Wellbeing and Preventable Chronic Disease Division, Menzies School of Health Research, Casuarina 0810, Australia; bvo3@une.edu.au (B.V.); PeterBaade@cancerqld.org.au (P.D.B.); Lisa.Whop@menzies.edu.au (L.J.W.); gail.garvey@menzies.edu.au (G.G.); 2 Cancer Research Centre, Cancer Council Queensland, Herston 4006, Australia; 3 University Centre for Rural Health, The University of Sydney, Lismore 2480, Australia; veronica.matthews@sydney.edu.au (V.M.); jodie.bailie@sydney.edu.au (J.B.); ross.bailie@sydney.edu.au (R.B.); 4 School of Population and Global Health, The University of Western Australia, Crawley 6009, Australia; barbara.nattabi@uwa.edu.au

**Keywords:** cervical cancer screening, continuous quality improvement, primary health care, Aboriginal and Torres Strait Islander, Indigenous, Australia, Pap smear, clinical audits, primary care, preventive health

## Abstract

Aboriginal and Torres Strait Islander women have significantly higher cervical cancer incidence and mortality than other Australian women. In this study, we assessed the documented delivery of cervical screening for women attending Indigenous Primary Health Care (PHC) centres across Australia and identified service-level factors associated with between-centre variation in screening coverage. We analysed 3801 clinical audit records for PHC clients aged 20–64 years from 135 Indigenous PHC centres participating in the Audit for Best Practice in Chronic Disease (ABCD) continuous quality improvement (CQI) program across five Australian states/territories during 2005 to 2014. Multilevel logistic regression models were used to identify service-level factors associated with screening, while accounting for differences in client-level factors. There was substantial variation in the proportion of clients who had a documented cervical screen in the previous two years across the participating PHC centres (median 50%, interquartile range (IQR): 29–67%), persisting over years and audit cycle. Centre-level factors explained 40% of the variation; client-level factors did not reduce the between-centre variation. Screening coverage was associated with longer time enrolled in the CQI program and very remote location. Indigenous PHC centres play an important role in providing cervical screening to Aboriginal and Torres Strait Islander women. Thus, their leadership is essential to ensure that Australia’s public health commitment to the elimination of cervical cancer includes Aboriginal and Torres Strait Islander women. A sustained commitment to CQI may improve PHC centres delivery of cervical screening; however, factors that may impact on service delivery, such as organisational, geographical and environmental factors, warrant further investigation.

## 1. Introduction

Since the introduction of Australia’s nationally organised cervical screening program (NCSP) in 1991, cervical cancer incidence and mortality rates have substantially decreased for Australian women [1,2]. During 1991 to 2014, the national incidence rate fell from 18 cases to 7 cases per 100,000 women and mortality fell from 4 to less than 2 deaths per 100,000 cases [1]. However, despite overall improvements, there are still large inequities in cervical cancer outcomes among some groups. Age-standardised incidence of cervical cancer is over twice as high for Aboriginal and Torres Strait Islander women compared to non-Indigenous women (15.9 vs. 6.3 per 100,000 women in 2009–2013, respectively) and mortality is almost four times as high (6.6 vs. 1.7 deaths per 100,000 in 2011–2015, respectively) [3]. In absolute terms, cervical cancer is the fifth most common cancer and the seventh most common cancer cause of death for Aboriginal and Torres Strait Islander women [3].

The NCSP offered biennial Papanicolaou (Pap) smear tests to women aged 18–69 years until recently [1]. In December 2017, the program was updated, offering women aged 25–74 five-yearly human papillomavirus (HPV) cervical screening tests [1]. Cervical screening tests are available at no cost for most Australian women through the universal health insurance scheme, Medicare; although providers may charge a low service fee. In the pre-2017 program, there was no mechanism to invite women for their first eligible screening test. However, once screened and registered on the then state-based register, women could be recalled for subsequent screening tests. In addition, some primary health care (PHC) centres have electronic information systems capable of inviting and/or recalling clients for cervical screening.

Cervical screening can prevent cervical cancer through detection of precancerous cervical abnormalities and reduce morbidity and mortality due to early detection of cancerous abnormalities [1]. Participation in cervical screening was 56% for all Australian women in 2015–2016 and this has been relatively consistent over time (peak: 64% in 1998–1999) [1]. There are no national estimates of screening participation for Aboriginal and Torres Strait Islander women. This is largely due to deficiencies in screening registry data [4]; however, population estimates for the state of Queensland suggest rates for Aboriginal and Torres Strait Islander women are much lower than for non-Indigenous women (2010–2011: 34% Aboriginal and Torres Strait Islander women vs. 56% for non-Indigenous women) [5].

Cervical screening is offered almost exclusively in the PHC setting in Australia, with data from the Australian Institute of Health and Welfare (AIIHW) National Key Performance Indicators for Aboriginal and Torres Strait Islander primary health care reports showing persistent and large variation in the delivery of cervical screening across PHC centres [6,7]. During 2016–2017, 27% of Aboriginal and Torres Strait Islander women aged 20–69 years who were regular clients at a PHC centre had a cervical screening test, with centre coverage rates ranging from 0% to just over 70% [6]. Similarly, during 2013–2014 the median coverage was 31%, with wide between-centre variation [7].

Service-level factors underlying this variation in cervical screening service delivery need to be better understood in order to identify service delivery improvements to increase participation in screening and cervical cancer outcomes for Aboriginal and Torres Strait Islander women. With sustained screening and HPV vaccination coverage, Australia is predicted to reach elimination targets by 2020 [8]. However, reaching elimination targets for Aboriginal and Torres Strait Islander women will be delayed if these programs are not meeting the needs of these women. This study uses data collected for a large-scale continuous quality improvement program (CQI) to identify factors associated with variation in cervical screening coverage between Indigenous PHC centres across Australia.

## 2. Materials and Methods

### 2.1. Study Context and Design

Data were collected through the Audit and Best Practice for Chronic Disease (ABCD) National Research Partnership (‘The ABCD Partnership’), a national CQI initiative that aimed to improve provision of PHC in Aboriginal and Torres Strait Islander communities. Herein, Indigenous PHC centres refer to community-controlled and government-managed health services whose service populations include a substantial proportion of Aboriginal and Torres Strait Islander people and which were established to meet the needs of this population.

The protocol and planning of the ABCD project have been described in previous studies [9,10,11]. Briefly, the ABCD Partnership used a CQI approach to enhance health outcomes by assisting Indigenous PHC centres to assess and improve their systems for delivery of best-practice care, typically on an annual basis. Participating centres received automated reporting, training, and site support for conducting audits according to standard protocols, which was provided by One21Seventy, a not-for-profit agency affiliated with the ABCD Partnership [12]. The clinical audit tools cover various aspects of PHC including chronic illness care, child health, maternal health, preventive care, rheumatic heart disease and mental health. In total, 175 Indigenous PHC centres across five Australian states and territories participated in the ABCD Partnership. Of these, 135 centres used the preventive health audit tool, which includes cervical screening, and data from these services is included in the current study.

### 2.2. Data Collection

Over the period 2005–2014, participating centres used the Preventive Care audit tool [9] at least once and de-identified CQI audit data were provided by these centres to the ABCD Partnership for analysis. The purpose of this audit tool was to determine whether recommended preventive services were delivered to clients in the previous 24 months. The audit tool and parameters of the outcome measures were developed by an expert working group and based on evidence and best-practice guidelines. Clients were included in the audit if they: (1) were aged between 15 and 64 years; (2) were resident in the community for at least six of the last 12 months; (3) had no diagnosis of diabetes, hypertension, coronary heart disease, chronic heart failure, rheumatic heart disease or chronic kidney disease; (4) were not pregnant or were less than 6 weeks postpartum at the time of audit; (5) had a registered attendance at the PHC in the previous 24 months. All clients from participating centres were included in the audit if there were less than 30 eligible clients at that centre, but a random sample of at least 30 were selected if there were more than 30 eligible clients. Based on these criteria, clinical audit data were initially available for 8150 female clients.

To align with the NCSP two-year recommended screening interval that was current at the time of the study [1], we aimed to quantify the proportion of women at each participating PHC centre who had biennial cervical screening tests. However, as records were de-identified, it was not possible to link individuals’ records across audits. This introduced the potential for double counting of the same women across successive audit periods. Subsequently, when a centre had two consecutive audits within a two-year period, we retained only data from the first audit (excluding 3681 related audit records from consecutive audits). The NCSP’s target age group at the time of the study was 20–69 years; subsequently, 640 audit records for PHC centre clients aged 15 to 19 years were excluded from the analysis. A further 28 women were excluded due to missing data. In total, 3801 female client records from 135 distinct Indigenous PHC centres were included in the final analysis.

### 2.3. PHC Centre Characteristics

Data collected about the centre characteristics included: year of audit, geographical location; jurisdiction; estimated number of Aboriginal and/or Torres Strait Islander people in the service area (hereafter referred to as population size); whether the centre was government-operated or community-controlled; and the total number of audits of the CQI program that the centre had participated in. The geographical location of each Indigenous PHC centre was categorized into very remote, remote and non-remote, using the Australian Standard Geographical Classification (AGSC) [13]. Population size was categorized as less than 500 people, 501–999 people, or equal to and more than 1000 people. Due to the small number of clients from some states and territories, this analysis does not report on screening by jurisdiction.

### 2.4. Client Characteristics

Client data included: age at the time of audit (in 5 year age groups); Indigenous status (‘Aboriginal and Torres Strait Islander’ and ‘non-Indigenous’); time between the audit date and the most recent PHC attendance (“<6 months” and “≥6 months”); and whether the client had been first seen by an Aboriginal Health Worker (AHW) or another health professional at their last attendance. Date of last attendance recorded refers to the last PHC centre visit irrespective of the reason for the visit. This variable is included in the multivariable analysis as an indicator of patient engagement with their primary health care, which has been used in previous ABCD analyses. For consistency and comparability across ABCD analyses, we used the same cut-point for this measure. The type of staff member seen as the first point of contact at the last attendance is an indicator of involvement of Aboriginal and Torres Strait Islander health workers in clinical processes.

### 2.5. Outcome Measure

Women were classified as screened if they had a PHC record of a cervical screening test (i.e., a Pap smear) in the two-year audit period; all other eligible women from participating centres were classified as unscreened in that period.

### 2.6. Statistical Analysis

Stata (version 15) software (StataCorp, College Station, TX, USA) was used to perform the statistical analysis. We used unadjusted logistic regression to calculate crude odds ratios to describe the association between each of the centre and client characteristics and cervical screening. We then used multilevel logistic regression models to quantify the independent associations of each characteristic after adjusting for other characteristics in the model. This approach takes the clustered nature of the data into account (i.e., clients are nested within PHC centres) [14]. A sequential approach was used to develop the multilevel models and assess the contribution of each level to the variance explained [14]. First, we fit the “null” model with no explanatory variables to quantify the between-centre variation (i.e., cervical screening and centre ID were included in the model only). Second, a centre-level model was created by adding measured PHC centre characteristics to the null model. Third, a full model, with both centre and client characteristics, was developed. Potential interactions among centre-level and client-level characteristics were added in the full model in a stepwise manner and their significance was assessed, based on effect size and *p*-value (<0.05). Effect size, namely the odds ratios, and confidence intervals were used to assess the importance of each variable in the model. The reduction in the total model variance due to the addition of client-level variables to the centre-level model was calculated using proportional change in variance (PCV) [15].

### 2.7. Ethics Approval

Ethical approval for the ABCD National Research Partnership was obtained from research ethics committees in each relevant Australian jurisdiction: Human Research Ethics Committee of the Northern Territory Department of Health and Menzies School of Health Research (HREC EC00153); Central Australian Human Research Ethics Committee (HREC-12-53); New South Wales Greater Western Area Health Service Human Research Committee (HREC/11/GWAHS/23); Queensland Human Research Ethics Committee Darling Downs Health Services District (HREC/11/QTDD/47); South Australian Aboriginal Health Research Ethics Committee (04-10-319); Curtin University Human Research Ethics Committee (HR140/2008); Western Australian Country Health Services Research Ethics Committee (2011/27); Western Australia Aboriginal Health Information and Ethics Committee (111-8/05); and University of Western Australia Human Research Ethics Committee (RA/4/1/5051).

## 3. Results

### 3.1. Primary Health Care Centre and Client Characteristics

Characteristics of the 135 participating PHC centres are described in Table 1. The PHC centres contributed records from multiple audits (between one and four), with a minimum of 37 participating centres in 2007–2008 and a maximum of 77 in 2011–2012. Most of the centres were located in very remote areas (73%) and in either Queensland (36%) or the Northern Territory (46%), were government-operated (73%), and had completed at least one follow-up audit after the baseline audit (49%).

In total, 3801 records of female clients were included in this analysis (Table 2). On average, each centre contributed 34 audit records over the study period (range: 3–89). The mean age of the women at their last attendance in the audit period was 34 years (standard deviation (SD): 11years) and 89% were identified as Aboriginal and Torres Strait Islander women. For their last PHC centre visit, 78% attended in the previous six months and 18% of women saw an AHW as their first contact person.

### 3.2. Cervical Screening

Overall, the average cervical screening coverage rate across the participating centres was 50 percent (interquartile range (IQR): 29–67%). During 2005 and 2014 there was no significant change in the median proportion of women screened and the wide variation in the documented cervical screening coverage was maintained. For each audit year, between zero and three centres had no screening tests documented (median 1.2).

Before adjustment for potential confounders, PHC governance structure, centre location remoteness, and service population size were associated with cervical screening (Table 1). Client characteristics significantly associated with having had cervical screening were women’s age and timing of the previous PHC attendance (Table 2).

### 3.3. Multivariate Analysis

The null sequential multilevel model demonstrated a significant variation in cervical screening service delivery between PHC centres (variance: 1.01; standard error (se) = 0.17). This variation reduced by 40% after adjusting for centre-level characteristics (0.61; se = 0.11) but the addition of client-level factors did not reduce the variance further (0.63; se = 0.12).

There appeared to be a dose–response relationship between the number of CQI audits completed and the proportion of clients screened (Table 1). Centres that had completed two or more audit cycles had a higher screening coverage than centres that had completed a baseline audit only. Primary health care centres in very remote locations had higher screening coverage, particularly for centres with service populations over 500 people; however, confidence intervals around the point estimates were wide (Table 1).

Women’s age at their last PHC centre visit was also associated with cervical screening, after adjusting for centre-level and other client-level factors (Table 2). Women age 25–29 years had the highest proportion screened and compared to this group, younger women (20–24 years) and older women, particularly those aged above 50 years, were less likely to have been screened in the previous 24 months.

## 4. Discussion

This study found wide variation across the participating PHC centres in the proportion of active female clients who had a cervical screening test in each two-year audit period. A high degree of between-centre variation has similarly been documented in other ABCD study investigating the variation in the delivery of other preventive care services [11,16], such as for sexually transmitted infections (STI) testing [17,18], cardiovascular care [19], and diabetes care [20]. There were a small number of the PHC centres in the current study that, for some of the years, had no documented delivery of cervical screening tests, but for half of the centres the coverage rates ranged between almost one third (29%) and two-thirds (67%) of the women who attended at least once in the past two years. The AIHW’s National Key Performance Indicator reports found similar between-centre variation in cervical screening coverage [6,7].

The variation in the delivery of cervical screening services across PHC centres in communities with predominately Aboriginal and Torres Strait Islander populations is an important finding. While Australia has among the lowest cervical screening incidence and mortality rates in the world, largely attributable to the NCSP, Aboriginal and Torres Strait Islander women have persistently higher rates of cervical cancer than other Australian women, with rates more similar to countries without organised screening programs. Given cervical screening is almost entirely offered through the PHC system in Australia, the identification of service factors associated with high and low PHC centre screening coverage provides opportunities to develop models of care and targeted service delivery improvement strategies that may reduce the prevailing inequities in cervical cancer in Australia.

Sustained participation in the CQI program appeared to be a key driver behind higher cervical screening coverage. Ongoing involvement in CQI has previously been associated with improved best-practice care related to diabetes [20], maternal health [21], child health [22], and general preventive health [23]. CQI offers access to service-level data, and the tools required to assess and reflect on this data, to inform locally relevant strategies for service delivery improvements. Length of time in the CQI program may also reflect other characteristics of the centre that are related to improved best-practice care. While the centre-level factors measured in this study accounted for a reasonable proportion of the between-centre variation (40%), a large degree of variation remained unexplained. Organisational factors (e.g., staffing, organisational structure), the nature and degree of community linkages, the availability of systems and infrastructure to support ongoing CQI, and broader policy, workforce and environmental factors are likely to influence both a centre’s capacity and capability to delivery healthcare services and their level of commitment to a CQI program [16,17,24]. The organisational structure and management style of the centre may play a particularly critical role in CQI participation. It has been reported that Indigenous PHC centres involved in the ABCD Partnership CQI program were more likely to have sustained participation in the program if, organizationally, the centre valued clinical data for service delivery improvement, believed necessary change was within their means, and had a whole-of-community approach to service delivery [25].

We were unable to explore how the staff profile of centres impacts upon cervical screening coverage. There is evidence that having female practitioners and trained AHWs on staff to advocate for and perform cervical screening tests for screen-eligible women leads to increased breast and cervical screening participation [26,27]. A shortage of female practitioners has previously been identified as a barrier to breast and cervical screening participation for Aboriginal and Torres Strait Islander women [26], as well as Indigenous women in other countries [28]. In the current study, Indigenous PHC centres in very remote areas tended to record higher proportions of cervical screening than for centres in other areas; consistent with findings from the AIHW’s *National Key Performance Indicator* reports [6,7]. There is opportunity to learn from these centres and further investigation to identify key enablers of cervical screening is warranted. Possible reasons for higher levels of screening in remote health centres includes the relatively large female workforce in remote regions of Australia [29], the utility of specific models that PHC centres in very remote areas have implemented in order to improve quality of care, and the different patterns of healthcare utilization-e.g., women in urban and regional areas may attend other services for their cervical screening while women in very remote areas may be more likely to rely on their local PHC centre for all their healthcare needs. As the majority of the PHC centres in this study were from very remote areas (73%), this may explain why the average cervical screening coverage rate was 50 percent across 2005–2014, compared to 31 percent in 2013–2014 among the centres involved in the *National Key Performance Indicator* report [7].

The fact that one quarter of the centres were screening over two-thirds of their client-base, indicates that there is the potential for Indigenous PHC centres to reach a high level of coverage for Aboriginal and Torres Strait Islander women. While data derived through the CQI program can guide the design and implementation of locally relevant strategies to improve the delivery of, and reduce inequalities in, cervical screening, data at a systems level is also necessary to monitor and evaluate program-level changes. Recent changes to the new NCSP include the introduction of a new HPV Cervical Screening Test, an increase to the screening interval (2 years to 5 years), and the option for in-clinic self-sampling, and each of these factors are likely to impact on cervical screening delivery and participation [1]. As Australia aims towards effective elimination of cervical cancer [8], population-level data to monitor trends and disparities will be critical if current inequities are to be reduced and not further widened [30]. Historically, monitoring cervical screening participation among Aboriginal and Torres Strait Islander women has been hindered by a lack of Aboriginal and Torres Strait Islander identification information on pathology report forms, which is the primary source of data for the jurisdictional registers [4]. While it remains unclear how the new NCSP and its national register will collect and record Aboriginal and Torres Strait Islander identification information, support for a structured CQI program is likely to lead to improvements in the delivery of evidence-based care. Additionally, aggregated CQI data from individual PHC centres can identify trends across areas and over time to inform broader health service planning and policy development [31]. Clearly, this is an important area that requires ongoing service innovation, evaluation, and actions to improve cervical screening, which requires both Aboriginal and Torres Strait Islander leadership and organisational capacity building [23].

Although there appears to be an inverse relationship between age and cervical screening, client-level factors did not account for additional between-centre variation. In this study, women aged 25–29 years were most likely to have had a screen in the two-year audit period. Similarly, a Queensland population-based study found that participation was highest among women aged 30 to 49 years, with participation decreasing with age for those aged 50 years and older [4]. In the broader population, the higher cervical screening participation rate among 30–49-year-olds may reflect more women being opportunistically screened when presenting for ante- and post-natal care. However, in the current study, pregnant women and those in the first six weeks post-partum were excluded from the study, and the reason for the increase in screening among the 25–29-year-olds is unknown and warrants further investigation.

In this study, Aboriginal and Torres Strait Islander women were as likely to be screened as non-Indigenous women. In interpreting these results, however, we need to be cautious, as the centres included in this study were those who voluntarily enrolled in the ABCD Partnership CQI program. It is plausible that these centres may differ from non-participating centres in terms of factors that impact on the delivery of cervical screening [25]. In addition, data derived from CQI may aid PHC centres develop locally relevant community engagement activities, particularly for under-screened clients. Encouraging and supporting CQI processes across all PHC centres in Australia may be one mechanism through which the predicted elimination of cervical cancer in this country is achievable for all Australian women.

This study aimed to identify service-level factors associated with variation in the delivery of cervical screening across Indigenous PHC centres and, for several reasons, the coverage rate across the participating centres cannot be directly compared to previously published population cervical screening participation rates. The participation by PHC centres in the ABCD Partnership was voluntary and selection of centres was not random [9]. As such, the data may not be representative of all Indigenous PHC centres in Australia, and findings may not reflect cervical screening participation for women who attend non-participating centres, other types of PHC centres, or who do not use PHC services. This is likely to at least partially explain why the PHC cervical screening coverage rate was higher in this study than what has been reported in previous AIHW *National Key Performance Indicator* reports [6,7]. This study excluded women who had major chronic conditions, were pregnant during the audit period, did not have stable residence within the community during the year prior to the audit, and who were not regular PHC clients (i.e., they had not attended a participating centre in the previous 24 months). These factors are likely to be associated with lower cervical screening participation and thus the exclusion of these women is likely to over-estimate the actual screening coverage within the communities included in this study. It is possible that the client eligibility criteria of the CQI program may have differentially excluded Indigenous and non-Indigenous women and, thus, direct comparison of these groups of women using these data are not advised.

Additionally, the data used for this analysis were-based solely on clinical records which could have underestimated actual services delivery. Failure to document services at health centres has been recognized in a previous study [32], although use of systems to standardised data collection improves data collection [33]. De-identified data was provided by participating centres, in accordance with ethical approvals. This meant that we were unable to link women across audit periods to assess the person-level change in screening participation over time and determine factors associated with screening regularity. We also had to exclude some annual audits to avoid the double counting of women; as the then-current recommendation was to screen biennially, a single Pap smear would be captured in two consecutive CQI audits. This loss in data may have meant some women were missed in our analysis.

While these data considerations limit our ability to generalize the cervical screening coverage rate to all Australian PHC centres, the participating health centres represent a reasonably large proportion of all Indigenous PHC centres, are from a variety of locations across Australia, and have provided the most detailed and most consistent wide-scale data for Indigenous PHC centres to date. As such, this dataset provides an important advance on existing evidence regarding he scope of variation in service delivery performance and the factors associated with this variation. There is growing evidence that cervical screening participation is lower for Aboriginal and Torres Strait Islander women than other Australian women [5], and that there is wide variation in the degree to which PHC centres offer this service [6,7]. New evidence from this study provides support for wide-scale CQI policy and may inform the development of targeted service delivery improvement strategies and models of care that are capable of reducing the disparities in cervical cancer screening and outcomes for Aboriginal and Torres Strait Islander women.

## 5. Conclusions

Cervical screening among women attending Indigenous PHCs is variable, with some centres performing above the national average. This variation provides an opportunity to better understand the drivers of higher levels of service delivery in cervical cancer prevention and, given the relatively high coverage in very remote locations, we may be particularly able to learn from the models of care of centres in these areas. Sustained engagement in CQI has emerged as a clear driver behind primary health service delivery, not only for cervical screening but for other services. To maximize the benefit of CQI programs, it is now necessary to understand how organisational, environmental and system level factors moderate the relationship between CQI participation and service delivery. Indigenous PHC centres play a critical role in delivering cervical screening to Aboriginal and Torres Strait Islander women, who experience a disproportionate burden of cervical cancer. Strategies for improving the delivery of cervical screening by Indigenous PHC centres should be devised by, and for, Aboriginal and Torres Strait Islander women.

## Figures and Tables

**Table 1:** Associations between Indigenous primary health care centre (*n* = 135) characteristics and cervical screening among female clients (*n* = 3801) ^1^.

Characteristic	Participating Centres *n* (%)	Unadjusted Odds Ratio ^3^	Adjusted Odds Ratio ^4^
		(95% CI)	(95% CI)
Audit Year			
2005–2006	40 (30) ^2^	1.00	1.00
2007–2008	37 (27)	1.40 (1.08–1.81)	0.96 (0.68–1.39)
2009–2010	45 (33)	1.04 (0.81–1.32)	0.68 (0.47–0.97)
2011–2012	77 (57)	1.51 (1.22–1.87)	0.88 (0.59–1.31)
2013–2014	56 (41)	1.26 (1.00–1.57)	0.65 (0.41–1.04)
		χ^2^(4) = 24, *p* < 0.001	
PHC Governance			
Community-controlled	37 (27)	1.00	1.00
Government	98 (73)	1.35 (1.17–1.56)	1.04 (0.69–1.56)
		χ^2^(1) = 18, *p* < 0.001	
Number of completed baseline and follow up audits			
Baseline audit	69 (51)	1.00	1.00
1–2 follow-up audits	21 (16)	1.33 (0.13–1.56)	1.63 (1.28–2.07)
≥3 follow-up audits	45 (33)	1.12 (0.96–1.31)	1.86 (1.35–2.57)
		χ^2^(2) = 13, *p* = 0.002	
Location Remoteness			Location (<500) ^5^
Non-remote	20 (15)	1.00	NR: 1.00
Remote	16 (12)	0.95 (0.73–1.25)	R: 0.30 (0.28–2.60)
Very remote	99 (73)	2.15 (1.77–2.62)	VR: 1.09 (0.11–10.70)
		χ^2^(2) = 105, *p* < 0.001	
			Location (501–999) ^5^
Service Population Size			NR: 1.00
<500	66 (49)	1.00	R: 0.97 (0.45–2.11)
501–999	24 (18)	0.82 (0.68–0.97)	VR: 2.44 (1.16–5.10)
≥1000	45 (33)	0.41 (0.36–0.48)	
		χ^2^(2) = 149, *p* < 0.001	Location (≥1000) ^5^
			NR: 1.00
			R: 1.55 (0.90–2.67)
			VR: 1.54(1.13–2.09)
Jurisdiction ^6^			
Northern Territory	62 (46)	1.93 (1.71–2.50)	-
Queensland	48 (36)	2.07 (1.61–2.32)	-
Other (NSW, WA, SA)	25 (19)	1.00	-
		χ^2^(2) = 64, *p* < 0.001	

Notes: Abbreviations: 95% CI: 95% confidence interval; NR: non-remote; R: Remote; VR: very remote; NSW: New South Wales; WA: Western Australia; SA: South Australia. ^1^ Women are classified as having received a cervical screening test if a Pap smear is documented in the previous two years. ^2^ The percentages reported here represent the proportion of the 135 participating PHC centres that contributed audit records in each audit year. As centres may have participated in multiple audits (up to four), the total number of centres across audit years totals more than 100%. ^3^ Odds Ratio (Yes: No) of women receiving a PHC record of a Pap test in previous two years. ^4^ Adjusted for all centre-level variables in Table 1 and client-level variables in Table 2 using a multilevel logistic regression model. ^5^ Significant interaction between Location and Service Area Population size (NR: Non-remote, R: Remote, VR: Very remote). ^6^ Due to small cell sizes, NSW, WA, and SA were grouped into ‘Other’ jurisdiction. Jurisdiction was not included in the multivariable model as collapsing the variable made it difficult to interpret.

**Table 2:** Associations between characteristics of women (*n* = 3801) attending Indigenous primary health care centres (*n* = 135) and cervical screening in the previous two years ^1^.

Characteristic	Women *n* (%)	Screened	Unadjusted Odds Ratio ^2^	Adjusted Odds Ratio ^3^
		%	(95% CI)	(95% CI)
Age (years)				
20–24	800 (21)	48	0.75 (0.61–0.93)	0.63 (0.50–0.80)
25–29	674 (18)	55	1.00	1.00
30–34	494 (13)	51	0.85 (0.67–1.07)	0.82 (0.63–1.06)
35–39	462 (12)	52	0.88 (0.69–1.11)	0.87 (0.67–1.14)
40–44	485 (13)	50	0.81 (0.64–1.03)	0.78 (0.60–1.02)
45–49	385 (10)	48	0.76 (0.59–0.98)	0.75 (0.56–0.99)
50–54	236 (6)	43	0.61 (0.46–0.83)	0.60 (0.43–0.84)
55–59	163 (4)	44	0.63 (0.45–0.90)	0.52 (0.35–0.87)
60–64	102 (3)	34	0.43 (0.28–0.66)	0.36 (0.22–0.59)
			χ^2^(8) = 26, *p* < 0.001	
Indigenous status ^4^				
Non-Indigenous	260 (7)	48	0.94 (0.73–1.21)	1.02(0.74–1.41)
Indigenous	3378 (89)	50	1.00	1.00
Not stated	163 (4)	37	0.60 (0.43–0.83)	0.80 (0.53–1.20)
			χ^2^(2) = 10, *p* = 0.007	
Time since last attendance				
≤6 months	2963 (78)	53	1.00	1.00
>6 months	838(22)	36	0.48 (0.41–0.57)	0.51 (0.42–0.61)
			χ^2^(1) = 83, *p* < 0.001	
Health professional seen at last attendance				
NDS	2963 (78)	50	1.00	1.00
AHW	685 (18)	47	0.87 (0.74–1.02)	0.96 (0.77–1.19)
Unknown	153 (4)	43	0.75 (0.54–1.04)	0.82 (0.53–1.26)
			χ^2^(2) = 5, *p* = 0.07	

Notes: Abbreviations: 95% CI: 95% confidence interval; NDS: Nurses, Doctors, Specialists or Other; AHW: Aboriginal Health Worker. ^1^ Women are classified as having received a cervical screening test if a Pap smear is documented in the previous two years. ^2^ Odds Ratio (Yes: No) of women receiving a PHC record of a Pap test in previous two years. ^3^ Adjusted for all centre-level variables in Table 1 and client-level variables in Table 2 using a multilevel logistic regression model. ^4^ We recommend caution in comparing Indigenous women to non-Indigenous women in this study due to the findings only being generalizable to women attending Indigenous PHC centres who voluntarily enrolled in the ABCD Partnership CQI program and a possible lack of broader documentation within records of clients with Indigenous status ‘not-stated’.

## Data Availability

The dataset analysed during the current study is not publicly available due to health centre confidentiality, but aggregated data may be available from the corresponding author on reasonable request and if consistent with the project’s ethics approvals.

## Abbreviations

ABCD	Audit and Best Practice for Chronic Disease
AHREC	Aboriginal Health Research Ethics Committee
AHW	Aboriginal Health Worker
CQI	continuous quality improvement
HREC	Human Research Ethics Committee
NCSP	National Cervical Screening Program
NRP	National Research Partnership
NSW	New South Wales
NT	Northern Territory
PCV	Proportional change in variance
PHC	primary health care
QLD	Queensland
SA	South Australia
WA	Western Australia
